# Long-Term Outcomes After Implantation of Magnesium-Based Bioresorbable Scaffolds—Insights From an All-Comer Registry

**DOI:** 10.3389/fcvm.2022.856930

**Published:** 2022-04-14

**Authors:** Matthias Bossard, Mehdi Madanchi, Dardan Avdijaj, Adrian Attinger-Toller, Giacomo Maria Cioffi, Thomas Seiler, Gregorio Tersalvi, Richard Kobza, Guido Schüpfer, Florim Cuculi

**Affiliations:** ^1^Cardiology Division, Heart Center, Luzerner Kantonsspital, Luzern, Switzerland; ^2^Faculty of Medicine, University of Zurich, Luzern, Switzerland; ^3^Department of Anaestesiology, Luzerner Kantonsspital, Luzern, Switzerland

**Keywords:** bioresorbable scaffold (BRS), Magmaris, percutaneous coronary intervention, scaffold thrombosis, stent, magnesium, outcome

## Abstract

**Background:**

The magnesium-based sirolimus-eluting bioresorbable scaffold (Mg-BRS) Magmaris™ showed promising clinical outcomes, including low rates of both the target lesion failure (TLF) and scaffold thrombosis (ScT), in selected study patients. However, insights regarding long-term outcomes (>2 years) in all-comer populations remain scarce.

**Methods:**

We analyzed data from a single-center registry, including patients with acute coronary syndrome (ACS) and chronic coronary syndrome (CCS), who had undergone percutaneous coronary intervention (PCI) using the Mg-BRS. The primary outcome comprised the device-oriented composite endpoint (DoCE) representing a hierarchical composite of cardiac death, ScT, target vessel myocardial infarction (TV-MI), and clinically driven target lesion revascularization (TLR) up to 5 years.

**Results:**

In total, 84 patients [mean age 62 ± 11 years and 63 (75%) men] were treated with the Mg-BRS devices between June 2016 and March 2017. Overall, 101 lesions had successfully been treated with the Mg-BRS devices using 1.2 ± 0.4 devices per lesion. Pre- and postdilatation using dedicated devices had been performed in 101 (100%) and 98 (97%) of all the cases, respectively. After a median follow-up time of 62 (61–64) months, 14 (18%) patients had experienced DoCEs, whereas ScT was encountered in 4 (4.9%) patients [early ScTs (<30 days) in three cases and two fatal cases]. In 4 (29%) of DoCE cases, optical coherence tomography confirmed the Mg-BRS collapse and uncontrolled dismantling.

**Conclusion:**

In contradiction to earlier studies, we encountered a relatively high rate of DoCEs in an all-comer cohort treated with the Mg-BRS. We even observed scaffold collapse and uncontrolled dismantling. This implicates that this metal-based BRS requires further investigation and may only be used in highly selected cases.

## Introduction

The latest generation of metallic drug-eluting stents (DESs) has largely reduced the risk of repeat revascularizations for target lesion failure (TLF) compared with bare-metal stents (1–3). However, metallic stents still bear the risk of late complications, including in-stent restenosis (ISR), neoatherosclerosis, and late stent thrombosis (4, 5).

Bioresorbable scaffolds (BRSs) have been developed to overcome some of the limitations seen in metallic stent platforms. They are intended to provide lumen patency in the early phase, allow vascular healing, and dissolve over time, thus potentially reducing the complications seen with a permanent metallic implant (6, 7). However, the mid- to long-term results of the first marketed BRS, the everolimus-eluting polymer poly-L-lactic acid (PLLA)-based BRS Absorb™ (Abbott Vascular, Santa Clara, California, USA), showed a high rate of device-related complications, particularly early and late scaffold thrombosis (ScT), which ultimately led to its withdrawal from the market (7–10).

In contrast, a series of studies indicated that the magnesium-based sirolimus-eluting BRS (Mg-BRS) Magmaris™ (Biotronik AG, Bülach, Switzerland) potentially represents a promising scaffolding technology for the treatment of acute and chronic coronary artery disease (CAD) (11–15). Its enhanced radial strength and lower tendency for device shortening might be important attributes that contribute to enhanced long-term results (16). However, its implantation also requires a meticulous approach encompassing adequate predilatation, sizing, and postdilatation (PSP) approach (17–19).

So far, a series of non-randomized, controlled studies, including the BIOTRONIKS – Safety and Performance in de NOvo Lesion of NatiVE Coronary Arteries With Magmaris (BIOSOLVE) I-IV trials, showed promising outcomes after treatment with the Mg-BRS, including low rates of target lesion failure (TLF) (<5%) and ScT (<1%) (11–15, 20, 21). But, there have also been published cases, which highlighted the collapse and early dismantling of this device lately (22, 23).

Overall, long-term outcome data (>2 years) after implantation of the Mg-BRS in an all-comer patient cohort that includes patients presenting with the acute coronary syndrome (ACS) and more complex lesions remain scarce. In this context, we analyzed outcomes and TLF patterns of consecutive patients included in the Mg-BRS all-comer registry.

## Methods

### Study Design and Patient Population

Between June 2016 and March 2017, we enrolled consecutive patients who underwent percutaneous coronary intervention (PCI) with implantation of the Magmaris™ Mg-BRS devices in our single-center MAGnesiuM-bAsed siRolImuS eluting, Bioresorbable Vascular Scaffold (MAGMARIS) registry. This registry was designed to assess the safety and performance of this device in a real-world setting. Those patients have also been included in the ongoing retrospective L-CAD registry, which was established to assess procedural characteristics and outcomes of patients requiring PCI for CAD.

We included patients presenting with both the acute and chronic CAD [including ST-segment elevation myocardial infarction (STEMI)], requiring stenting of *de-novo* lesions or ISR. This study was conducted at the Luzerner Kantonsspital, Lucerne, Switzerland, which represents the tertiary cardiology facility for the central part of Switzerland (PCI volume of ~1,800 cases/year). The registry was coordinated by the Research Division of Lucerne Heart Center, Switzerland. Patients were retrospectively consented to and underwent prospective follow-ups (office visits, phone calls, or chart reviewing). Both the registries have been approved by the Ethikkommission Nordwest- und Zentralschweiz (EKNZ; MAGMARIS registry: EKNZ; study ID: 2018-01036; and L-CAD registry: BASEC ID 2019-01067).

### Study Device and Implantation Technique

The Magmaris™ Mg-BRS (Biotronik AG, Bülach, Switzerland) represents a balloon-expandable, sirolimus-eluting bioresorbable metal scaffold on a rapid delivery system (24). The device consists of a fully bioresorbable magnesium alloy, which is expected to be resorbed by ~95% after 12 months. The struts are 150 μm thick, have a width of 150 μm, and are laser-polished. Their surface is completely coated with bioresorbable PLLA, which releases sirolimus. The scaffold is currently available in diameters of 3.0 and 3.5 mm and lengths of 15, 20, and 25 mm.

The decision to use this device was at the discretion of the operating physician. For implantation, we followed the manufacturer's and experts' recommendations, which mandated appropriate predilatation, preferably with a non-compliant balloon (NCB), sizing, and postdilatation (24). In selected cases (e.g., calcified lesions), the interventionalist might have decided to perform a “hybrid lesion treatment,” meaning combined implantation of the Mg-BRS and contemporary DES, to adequately cover the diseased segments.

After the device implantation, we ensured that all the patients received guideline-based dual antiplatelet therapy (DAPT), meaning aspirin plus clopidogrel, combined with either ticagrelor or prasugrel for at least 12 months. In patients who required anticoagulation, we recommended a direct oral anticoagulant in combination with aspirin for 7–28 days and clopidogrel for 12 months.

Follow-ups were conducted by phone calls, clinical visits, and/or chart reviewing at 6 months, 1 year, 2 years, and up to 5 years after scaffold implantation.

### Outcome Definitions

Our primary clinical endpoint consisted of a device-oriented composite endpoint (DoCE) reflecting a hierarchical composite of cardiac death, ScT, target vessel myocardial infarction (TV-MI), and clinically driven target lesion revascularization (TLR) up to 5 years (11). Cardiac death, clinically driven TLR, and ScT were defined as suggested by the Academic Research Consortium (ARC) criteria (25, 26). The secondary endpoints of interest were target-vessel revascularization (TVR) and coronary artery bypass graft (CABG) surgery. In addition, we collected information regarding the occurrence of non-cardiovascular death. For MI, we applied the universal definition (27). ScT was classified as definite, probable, and possible (25, 26). Accordingly, the presence of a thrombus that occurred within the scaffold or its 5 mm proximal or distal margins on angiographic or intravascular imaging and the presence of either: (1) acute onset of ischemic symptoms, (2) novel electrocardiographic changes that implicated ischemia, (3) a rise and fall in cardiac biomarkers, or a combination of the latter defined ScT. Timing of ScT was categorized according to the ARC criteria for stent thrombosis as early (<30 days after implantation), late (>30 days to 1 year after implantation), and very late (>1 year after BRS implantation) (25, 26). All the angiograms, optical coherence tomography (OCT) investigations, and clinical outcomes were reviewed and adjudicated by two independent cardiologists not involved in the enrollment and device implantation (MB and GMC).

### Optical Coherence Tomography Acquisition and Analysis

In case of DoCE, our BRS follow-up protocol suggests obtaining an OCT that was acquired before and ideally after PCI. For OCT, we used the Optis Ilumien™ System and the Dragonfly Duo OCT Imaging Catheter (St Jude Medical/Abbott, Minneapolis, Minnesota, USA) with motorized pullback (25 mm/s) using a non-occlusive flushing technique according to the manufacturer's recommendations. Images of the scaffold and of the reference segments 10 mm proximal and distal of the scaffold were acquired. OCT pullbacks were registered and assessed offline using dedicated software (Lightlab Imaging, St Jude Medical, Minnesota, USA). We applied the same methodology and definitions, as described elsewhere earlier (26).

### Statistical Methodology

We did not prespecify a sample size for enrollment, since this registry was designed as an observational study to analyze early- and long-term outcomes after the Mg-BRS implantation. Statistical analyses were primarily descriptive. Categorical variables are displayed as absolute numbers and percentages. Continuous variables are presented as means (±SDs) or medians [interquartile ranges (IQRs)], as appropriate. *P*-values were calculated using the paired *t*-tests, Fisher's exact test, and the chi-squared test, where applicable. The Kaplan–Meier curves were plotted to assess the distribution of adverse clinical outcomes over time. The univariate and multivariate Cox regression models were used to assess the possible predictors for the primary study endpoint DoCE. Parameters reaching a *p*-value < 0.05 in the univariate model were further subjected to the multivariate model. *p* ≤ 0.05 was considered as statistically significant. All the analyses were conducted using STATA version 16 (College Station, Texas, USA).

## Results

### Patients and Treated Lesions

We treated 84 patients with at least one Mg-BRS device at our institution. Mean age was 62 ± 11 years and 63 patients (75%) were male. Overall, 56 (66 %) patients presented with an ACS, and 28 (34 %) patients underwent PCI for chronic coronary syndrome (CCS). Baseline characteristics are shown in Table 1.

**Table 1:** Baseline characteristics grouped according to outcomes during follow-up.

	**Overall** **(*n* = 84)**	**No DoCE** **(*n* = 70)**	**DoCE** **(*n* = 14)**	***P*** -value^*^
Age (years ± SD)	62 ± 11	62 ± 11	63 ± 6	0.43
Males, *n* (%)	63 (75)	50 (71)	13 (93)	0.09
Presentation, *n* (%)				0.71
CCS	28 (34)	22 (31)	6 (43)	
UA/ NSTEMI	34 (40)	29 (41)	5 (36)	
STEMI	22 (26)	19 (27)	3 (21)	
Heart rate (bpm ± SD)	73 ± 16	71 ± 16	82 ± 17	0.01
Systolic blood pressure (mmHg ± SD)	115 ± 21	115 ± 20	119 ± 22	0.27
Diastolic blood pressure (mmHg ± SD)	72 ± 16	71 ± 16	76 ± 8	0.14
Left ventricular ejection fraction, *n* (%)	57 ± 10	57 ± 10	57 ± 11	0.48
Creatinine (mmol/L ± SD)	83 ± 23	83 ± 25	79 ± 10	0.31
Arterial hypertension *n* (%)	49 (58)	40 (57)	9 (64)	0.62
Diabetes mellitus *n* (%)	10 (12)	8 (11)	2 (14)	0.76
Dyslipidemia, *n* (%)	44 (52)	35 (50)	9 (64)	0.33
Current smoking, *n* (%)	43 (51)	35 (50)	8 (57)	0.62
Family history of premature CAD, *n* (%)	24 (29)	19 (27)	5 (36)	0.52
Previous MI, *n* (%)	19 (23)	15 (21)	4 (29)	0.56
Previous CABG, *n* (%)	3 (3.6)	2 (2.9)	1 (7.1)	0.43
Antithrombotics, *n* (%)				
Aspirin	83 (99)	69 (99)	14 (100)	-
Clopidogrel	36 (43)	30 (43)	6 (43)	1.00
Ticagrelor	33 (39)	27 (39)	6 (43)	0.76
Prasugrel	14 (17)	13 (19)	1 (7.1)	0.29
Direct oral anticoagulant	3 (3.6)	2 (2.9)	1(7.1)	043
GPIIbIIIa inhibitor	41 (49)	33 (47)	8 (57)	0.49
Access, *n* (%)				0.32
Radial	72 (86)	62 (89)	10 (71)	
Femoral	12 (14)	9 (13)	3 (21)	

*Data are mean (standard deviation) or number (percentage), as appropriate*.

*Bpm, Beats per minute; CCS, chronic coronary artery syndrome; CAD, Coronary artery disease; CABG, Coronary artery bypass grafting; DoCE, device-oriented composite endpoint; GPIIbIIIa, Glycoprotein IIbIIIa; MI, Myocardial infarction; NSTEMI, Non-ST-segment elevation myocardial infarction; STEMI, ST-segment elevation myocardial infarction; UA, Unstable angina*.

* *P-values were based on student's t-tests, Fisher's test or Chi-square tests, as appropriate*.

In total, 101 lesions were treated and all the devices were successfully implanted by angiographic means. The most treated vessel was the left anterior descending (LAD) artery (49%) and type B2/C lesions represented one-third of all the lesions. Eight and 3% of all the lesions were ISR and CTO lesions, respectively. Mean device diameter was 3.25 ± 0.25 mm and mean device length was 22.1 ± 3.3 mm. All the lesions were predilated. Postdilatation was performed in 98 (98%) lesions, predominantly using the super high-pressure NCB OPN™ balloon (SIS Medical, Frauenfeld, Switzerland), applying a mean pressure of 32 ± 8 atm. Further details focusing on lesion and implantation characteristics are given in Table 2.

**Table 2:** Lesion characteristics grouped according to outcomes during follow-up.

	**No. lesions** **(*n* = 101)**	**No DoCE** **(*n* = 87)**	**DoCE** **(*n* = 14)**	***P*** **-value^†^**
Vessels treated, *n* (%)				0.80
Left anterior descending	49 (49)	45 (49)	6 (43)	
Left circumflex	23 (24)	19 (22)	4 (29)	
Right coronary artery	27 (27)	23 (26)	4 (29)	
Lesion types, *n* (%)				0.96
A	25 (25)	21 (24)	4 (29)	
B1	41 (41)	35 (40)	6 (43)	
B2	30 (30)	26 (30)	4 (29)	
C	5 (5)	5 (2.8)	0 (0)	
ISR, *n* (%)	8 (8)	6 (6.9)	2 (14)	0.34
Bifurcation lesions, *n* (%)	16 (16)	13 (15)	3 (21)	0.54
Aorto-ostial lesions, *n* (%)	0 (0.0)	0 (0)	0 (0)	-
CTO, *n* (%)	3 (3)	3 (3.4)	0 (0)	-
Degree of calcification, *n* (%)				0.92
None	45 (38.3)	39 (45)	6 (43)	
Mild-to-moderate	51 (50)	44 (51)	7 (50)	
Severe	5 (5)	4 (4.6)	1 (7.1)	
Initial TIMI flow, *n* (%)				0.28
0	30 (30)	27 (31)	3 (21)	
1	2 (2)	1 (1.1)	1 (7.1)	
2	5 (5)	5 (5.7)	0 (0.0)	
3	64 (63)	54 (62)	10 (71)	
Final TIMI flow, *n* (%)				-
2	2 (2.0)	2 (2.3)	0 (0.0)	
3	99 (98)	85 (98)	14 (100)	
No. of Mg-BRS per lesions, *n* (%)	1.2 ± 0.4	1.72 ± 0.38	1.21 ± 0.41	0.35
Mean device diameter (mm ± SD)	3.25 ± 0.25	3.25 ± 0.25	3.21 ± 0.25	0.30
Mean device length (mm ± SD)	22.1 ± 3.3	22.2 ± 3.3	21.7 ± 3.5	0.29
Deployment pressure (mm ± SD)	13.7 ± 2.5	13.5 ± 2.5	14.6 ± 2.2	0.07
Hybrid lesion treatment, *n* (%)	28 (8)	24 (28)	4 (20)	0.94
Pre-dilatation, *n* (%)	101 (100.0)	87 (100.0)	14 (100.0)	-
Pre-dilatation device, *n* (%)				0.35
SC-balloon	10 (10)	10 (12)	0 (0.0)	
NC- balloon	91 (90)	77 (89)	14 (100)	
Post-dilatation, *n* (%)	98 (97)	84 (97)	14 (100)	1
Post-dilatation device, *n* (%)				0.20
SC- balloon	5 (5.1)	5 (6)	0 (0.0)	
NC- balloon	41 (42)	37 (44)	4 (29)	
Super-NC balloon	52 (53)	42 (50)	10 (71)	
Maximal mean postdilatation pressure (atm ± SD)	24.2 ± 7.3	24.0 ± 7.1	25.4 ± 8.2	0.27
Fenestration of side branch, *n* (%)	11 (11)	11 (10.2)	0 (0.0)	-
Intravascular imaging guidance, *n* (%)	8 (7)	8 (9.2)	0 (0.0)	0.6

*Data are mean (standard deviation) or number (percentage), as appropriate*.

*CTO, chronic total occlusion; DoCE, device-oriented composite endpoint; ISR, in-stent restenosis; Mg-BRS, Magnesium bioresorbable scaffolds; NC, non-compliant balloon; No., Number; SC, semi-compliant balloon; TIMI, Thrombolysis in Myocardial Infarction*.

† *P-values were based on student's t-tests, Mann-Whitney U- tests, or Chi-square tests, as appropriate*.

### Clinical Outcomes

**Figure 1:**
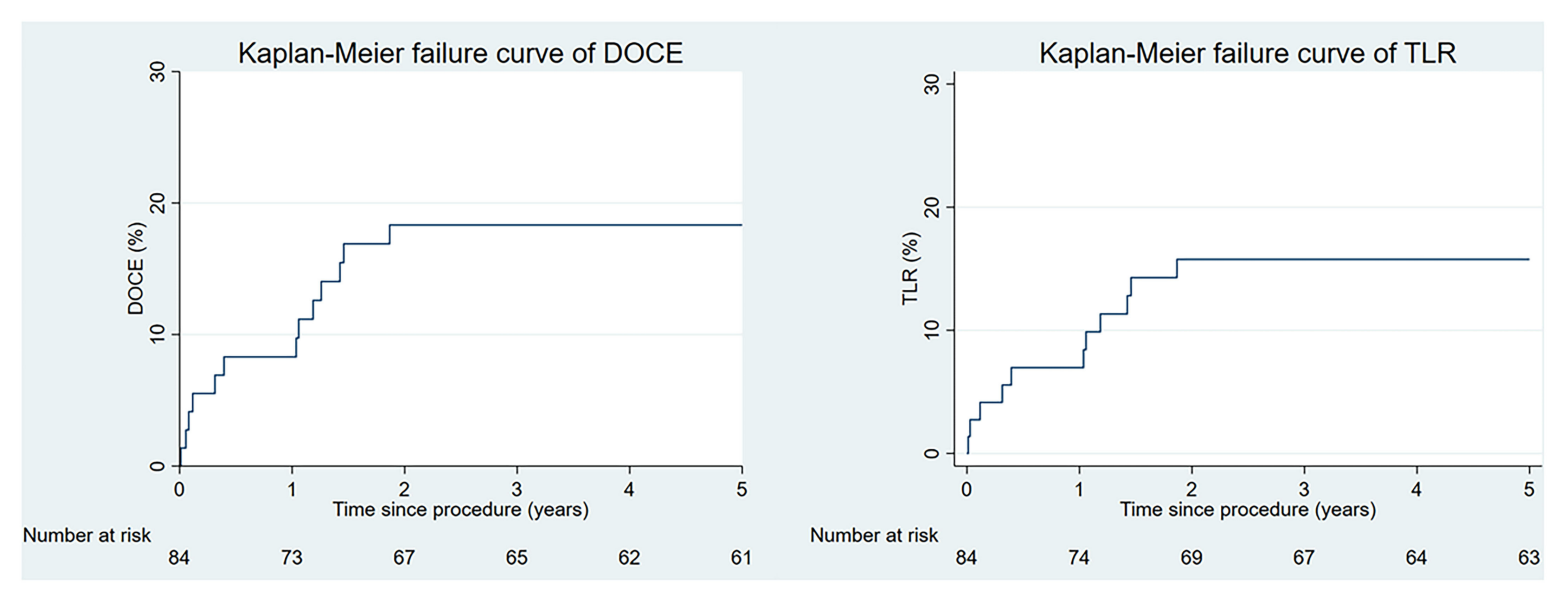
The Kaplan–Meier curves for the device-oriented composite endpoint (DoCE) and target lesion revascularization (TLR) over time.

Complete clinical follow-up was achieved in 72 (86%) patients at a median (IQR) duration of 62 (61–64) months. DoCE occurred in 14 patients, reaching 18% at 5 years. Clinically driven TLR represented the driving cause of DoCE reaching 16% at 5 years. The distribution of the main adverse events is given in Figure 1. Clinical outcomes are shown in Table 3, whereas the narratives of DoCE are shown in Table 4.

**Table 3:** Clinical outcomes up to 5 years follow-up.

**Clinical outcomes**	**6 months**	**1 year**	**2 years**	**3 years**	**4 years**	**5 years**
**Patients at follow-up, *n* (%)^†^**	**83 (99)**	**80 (95)**	**78 (94)**	**75 (89)**	**72 (86)**	**72 (86)**
**Primary endpoint *n* (%):**						
DoCE^*^	6 (7.2)	8 (9.7)	13 (16)	13 (18)	13 (16)	14 (18)
Cardiac death	1 (1.2)	1 (1.2)	2 (2.5)	3 (3.6)	3 (3.6)	3 (3.6)
ScT	3 (3.6)	4 (4.9)	4 (4.9)	4 (4.9)	4 (4.9)	4 (4.9)
TV-MI	3 (3.6)	4 (4.9)	4 (4.9)	4 (4.9)	4 (4.9)	4 (4.9)
TLR	5 (6.1)	7 (8.5)	11 (14)	11 (14)	11 (14)	12 (16)
**Secondary endpoints, *n* (%):**						
TVR	2 (2.5)	2 (2.5)	3 (3.9)	3 (3.9)	3 (3.9)	7 (11)
CABG	0 (0.0)	0 (0.0)	0 (0.0)	2 (2.5)	2 (2.5)	5 (6.1)
**Non-cardiac death, *n* (%)**	0 (0.0)	3 (3.6)	4 (4.9)	4 (4.9)	4 (4.9)	4 (4.9)

*Data are presented as number (percentage) and represent Kaplan-Meier estimates*.

*CABG, Coronary artery bypass grafting; DOCE, device-oriented composite endpoint; ScT, scaffold thrombosis; TLR, target lesion revascularization; TV-MI, target vessel myocardial infarction; TVR, target vessel revascularization*.

* *DoCE (device-oriented composite endpoint) represents a hierarchical composite of cardiac death, scaffold thrombosis (ScT), target vessel myocardial infarction (TV-MI), and clinically driven target lesion revascularization (TLR). Technically, only one adverse outcome of this composite was counted*.

† *A total of seven patients died, and five patients were lost to follow-up (two of them left Switzerland and three withdrew from the study during follow-up)*.

**Table 4:** Narratives of the 14 patients with a DoCE.

**DoCE no**.	**Time to DoCE (days)**	**DoCE presentation**	**Presumed cause of DoCE**	**Indication for index PCI**	**Targeted vessels**	**Lesion with complex features**	**Target vessel**	**No. of BRS in target lesion**	**BRS diameter (mm)**	**BRS length (mm)**	**P2Y12 inhibitor**
1	4	STEMI	Early ScT, presumably undersized scaffold	NSTEMI (UA)	1	BL (0,1,1)	Mid LAD	2	3.0	25	Clopidogrel
2	20	STEMI	Early ScT, premature discontinuation of DAPT^*^	CCS (angina)	1	BL (1,0,0)	Distal RCA	1	3.0	25	Ticagrelor
3	29	STEMI	Early ScT due to device collapse	CCS (staged PCI)^†^	1	No	Mid LAD	1	3.5	25	Ticagrelor
4	42	Elective PCI^‡^	Device dismantling and collapse with RST	NSTEMI	1	BL (1,0,1)	Proximal LAD	1	3.5	25	Ticagrelor
5	113	Stable angina	Device dismantling and collapse with RST	CCS (angina; Instent-Restenosis)	1	ISR/ BL (0,0,1)	Proximal LAD	1	3.0	20	Clopidogrel
6	142	NSTEMI	Device dismantling and collapse with RST	CCS (angina)		No	Proximal LAD	2	3.5 / 3.0	20 / 25	Clopidogrel
7	373	UA	Restenosis after scaffold dissolution	NSTEMI	1	No	Proximal LCx	1	3.0	20	Ticagrelor
8	381	STEMI	Very late ScT	NSTEMI	1	BL (1,0,0)	Proximal LAD	2	3.5	25 / 20	Ticagrelor
9	427	Stable angina	RST	CCS (angina)	1	ISR	Mid RCA	2	3.25	25	Clopidogrel
10	453	CV death	-	NSTEMI	-	No	-	-	-	-	-
11	513	UA	Device collapse and floating struts	NSTEMI (UA)	1	No	Proximal LAD	1	3.5	25	Prasugrel
12	525	NSTEMI	RST	NSTEMI	1	No	Mid RCA	2	3.5	20	Clopidogrel
13	672	STEMI	RST	CCS (angina)	1	No	Proximal to mid RCA	3	3.0/3.5/3.5	25/25/15	Ticagrelor
14	1,835	Stable angina	RST	CCS (angina)	1	No	Distal LAD	1	2.5	25	Clopidogrel

*BL, bifurcation lesion; BRS, bioresorbable scaffold; CCS, Chronic coronary syndrome; CV-death, cardiovascular death; DAPT, Dual antiplatelet therapy; DoCE, device-oriented composite endpoint; NSTEMI, Non-ST-segment elevation myocardial infarction; LAD, Left anterior descending coronary artery; LCX, Left circumflex coronary artery; PCI, Percutaneous coronary intervention; RCA, Right coronary artery; RST, Re-stenosis; ScT, Scaffold thrombosis; STEMI, ST-segment myocardial infarction; UA, Unstable angina*.

* *Ticagrelor was paused prior to minimal invasive CABG surgery*.

† *This patient had a staged PCI of a significant proximal LAD lesion after an inferior STEMI requiring RCA PCI*.

‡ *Patient underwent elective PCI of the left circumflex. While doing so, Mg-BVS dismantling and collapse resulting in re-stenosis within the LAD was found (on OCT)*.

Of note, ScT was encountered in four patients (4.9%) at a median time of 24 (IQR 12–205) days. Three ScTs were definitive (Supplementary Figure 1), whereas 1 ScT was probable. *Early ScT* (<30 days post-implantation) occurred in 3 (3.6%) patients (4, 20, and 29 days after implantation), which all presented with STEMI. *Very late ScT* (>1 year after implantation) occurred in 1 patient (381 days after implantation) presenting with non-STEMI (NSTEMI). Supplementary Figure 2 depicts the angiographic presentation of a patient with an early ScT.

### Optical Coherence Tomography Findings in Patients With DoCE

**Figure 2:**
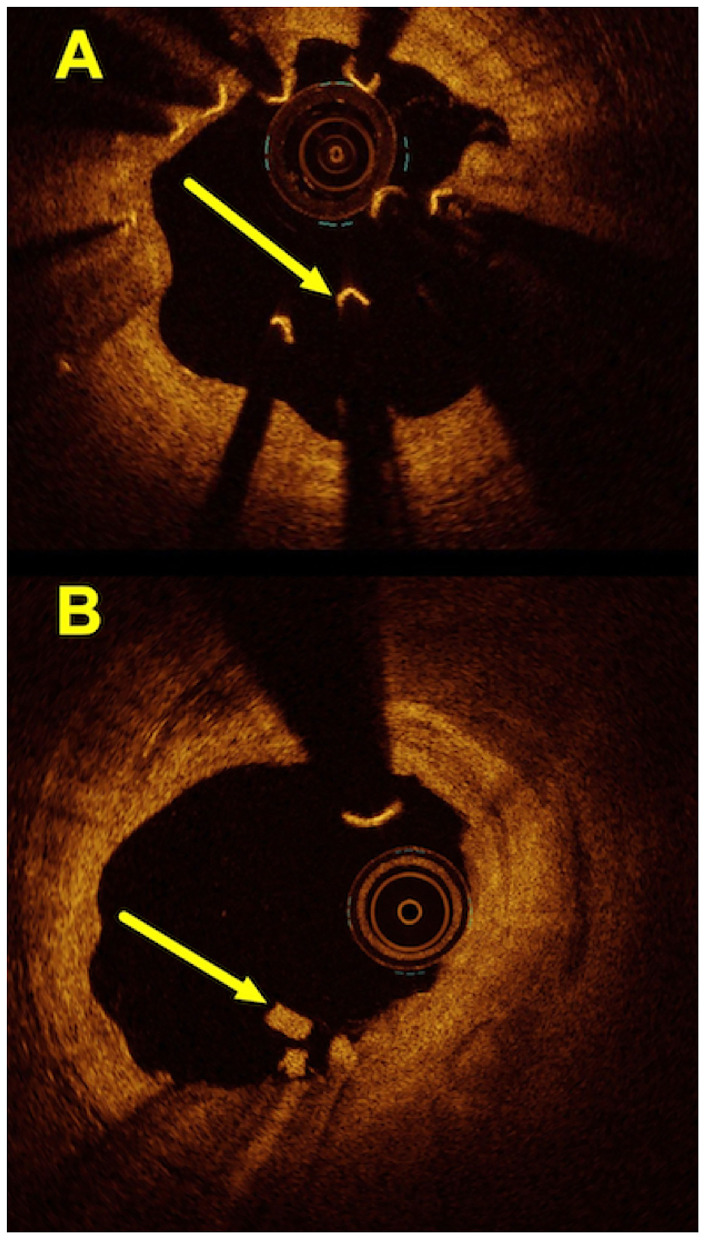
Hallmark OCT findings encountered in patients with the Mg-BRS-related target lesion failures: **(A)** scaffold collapse; **(B)** uncontrolled device dismantling. **(A)** This OCT image shows a collapsed scaffold with seemingly “free-floating” struts (*arrow*), which are no longer embedded and apposed to the coronary artery's wall. Also, the inner lumen appears to have restenosis with significant luminal irregularities and tissue protrusion. **(B)** This illustrates an incompletely dismantled Mg-BRS with residuals of struts protruding into the vascular lumen (*arrow*). Also, the intima appears partially irregular (1–5 o'clock). Mg-BRS, magnesium-based sirolimus-eluting bioresorbable scaffold; OCT, optical coherence tomography.

**Figure 3:**
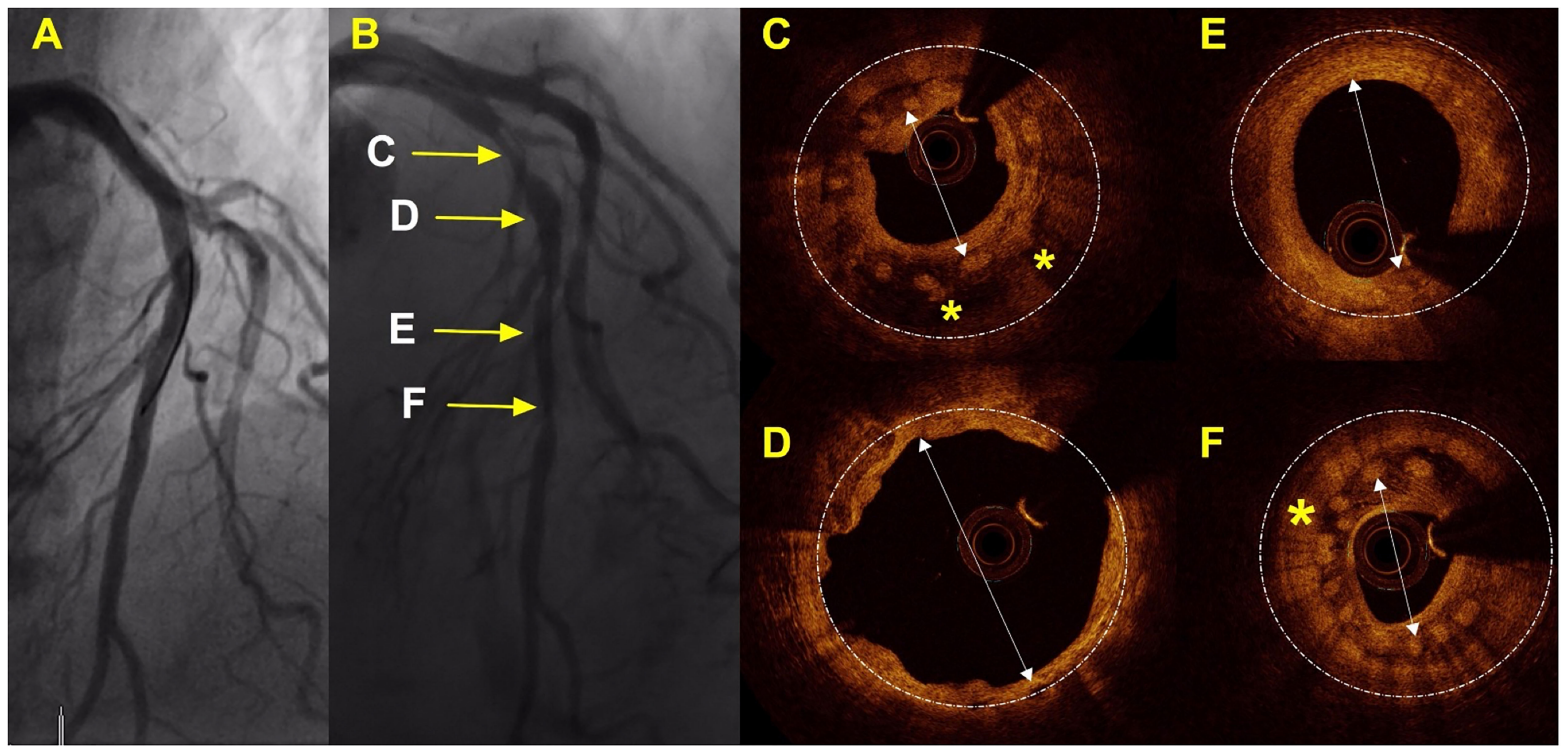
Case with TLR 42 days after implantation of the 2 Mg-BRS. Angiographic and OCT results of a case that required TLR. **(A)** By angiographic measures, a good result was achieved after implantation of the 2 Mg-BRS devices to the left anterior coronary artery; **(B)** This shows the angiography of the left anterior descending artery 42 days after the scaffold implantation, including serial segments **(C,E,F)** with significant luminal narrowing [>75% by Quantitative Coronary Angiography (QCA)]; **(C,F)** These images illustrate device collapse and uncontrolled dismantling associated with excessive tissue formation and significant lumen loss; **(D)** This segment shows eccentric remodeling of the artery wall after complete degradation of the Mg-BRS; **(E)** On OCT, the Mg-BRS seems completed degraded in this segment of left anterior descending (LAD). Mg-BRS, magnesium-based sirolimus-eluting bioresorbable scaffold; OCT, optical coherence tomography. ^*^: denotes the scaffold and arrow: denotes the vessel lumen.

**Figure 4:**
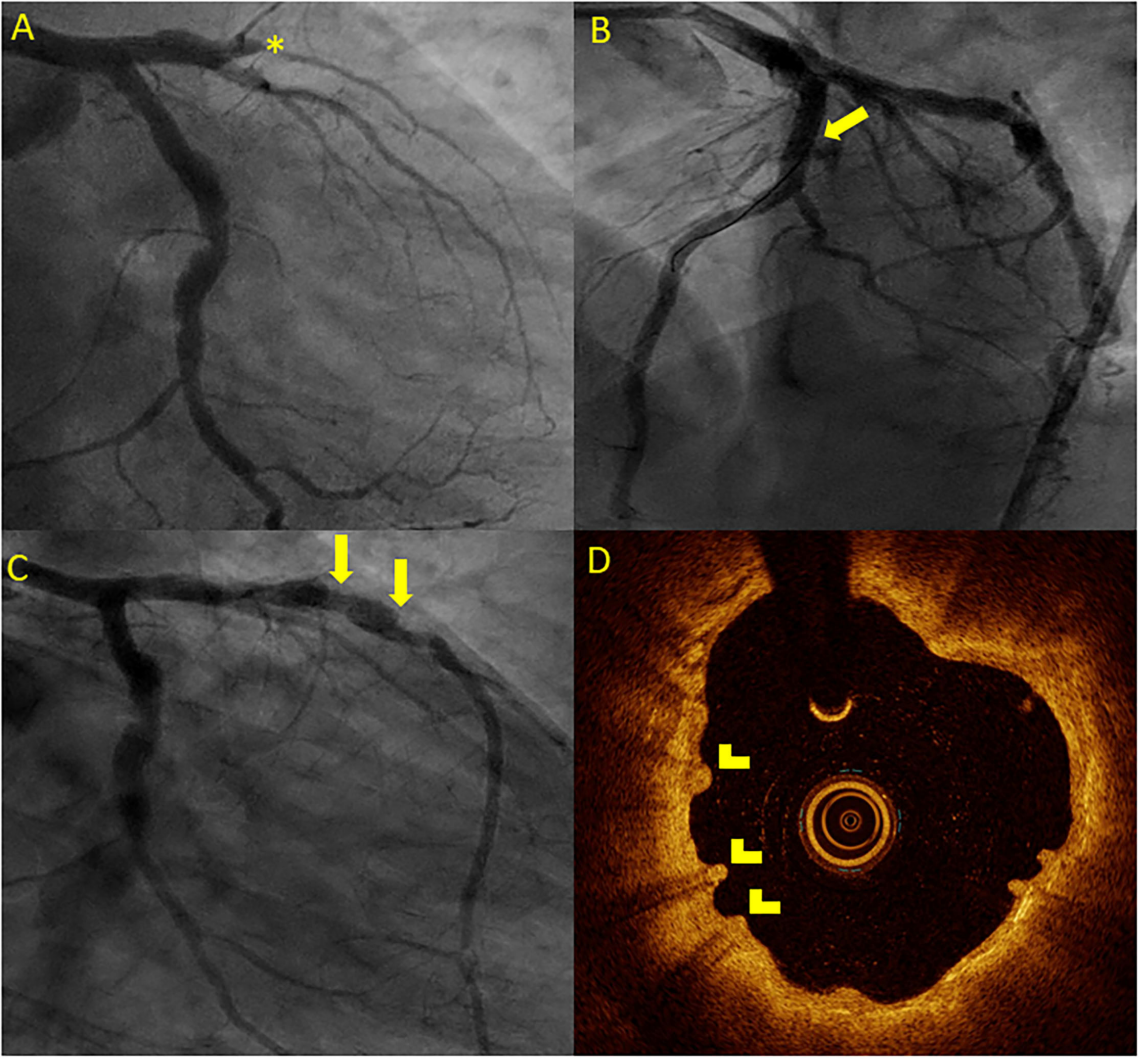
Case vignette of a patient treated with the 2 Mg-BRS, depicting long-term scaffold persistence and negative vessel remodeling. A 46-year-old patient presented to the emergency department with an acute anterior ST-segment elevation myocardial infarction (STEMI). On the coronary angiogram, a complete occlusion of the mid LAD artery was observed **(A)**. After predilatation with a non-compliant balloon (2.5 mm × 20 mm, 14 atm), the 2 Magmaris BRS (3 mm × 25 mm and 3.5 mm × 25 mm) were successfully implanted **(B)**. The patient presented 4 years later with chest pain during physical activity [chronic coronary syndrome II (CCS II)]. On the coronary angiogram **(C)**, we found an aneurysmatic proximal to mid LAD without relevant stenosis. On OCT **(D)**, scaffold remnants were observed as an expression of incomplete scaffold degradation. ^*^: complete vessel occlusion, arrow: coronary aneurysma, and arrowhead: scaffold remnants.

Overall, 7 (50%) patients with a DoCE had OCT imaging. The hallmark OCT findings encountered among patients with TLF are given in Figures 2, 3. Additionally, we encountered 1 case of scaffold persistence 5 years after implantation as described in the case vignette (Figure 4).

## Discussion

Bioresorbable scaffolds were received with a lot of enthusiasm from the cardiology community, since the concept itself seems appealing for the treatment of CAD. A scaffold, which provides radial strength in the acute phase and dissolves over time, has the potential to reduce long-term complications seen with DES (18, 28). However, this early euphoria has been curbed by the rather discouraging results seen with the Absorb™ BRS (8–10, 29). Nonetheless, the concept of scaffolding coronary artery lesions did not completely vanish. In fact, there are still some devices commercially available, including the metal-based Magmaris™ BRS and a series of novel devices currently under investigation (30, 31). In this context, our long-term outcome data after implantation of the Magmaris™ BRS in an unselected patient cohort are important.

Our results derive from a well-characterized real-world population, including patients with MI and complex lesions and they stand in contrast to most of the other clinical studies assessing the performance of the Mg-BRS Magmaris™. The main findings of our long-term study (>2 years) are the following: first, we found a rather high rate of DoCE, reaching 18% at 5 years follow-up. Interestingly, the majority of adverse outcomes have not been observed within 12 months after the Mg-BRS implantation; second, we encountered DoCE in multiple patients, despite following vigorously the proposed PSP approach for scaffold implantations; finally, we also found hints for scaffold collapse, scaffold dismantling, excessive negative remodeling with scaffold remnants, and formation of aneurysms. To the best of our knowledge, this represents one of the longest follow-up studies of patients, which had been treated with the Mg-BRS.

To put our results into perspective, most of the published studies highlighted the safety and performance of the Magmaris™ scaffold (11–15, 20, 21, 32). The most recent BIOSOLVE-IV study showed excellent device and procedure success as well as remarkably good outcomes up to 24 months in a low-risk population (13, 32). However, the BIOSOLVE-IV trial's population and lesion characteristics differed from our study cohort (given in Supplementary Table 2), which potentially explain some disparities among the observed clinical outcomes. For instance, we enrolled more patients with ACS (66% in this study vs. 19% in BIOSOLVE-IV) and our lesions managed with the Mg-BRS appeared more complex (35 vs. 15% being type B2/C lesions and 16 vs. 5% involving bifurcation lesions). Notably, the median follow-up time of the BIOSOLVE IV trial was also shorter compared to this study (24 vs. 62 months). It is, thus, possible that the number of adverse events occurring in the BIOSOLVE IV cohort could further grow over time.

It has been suggested that the relatively thick struts of the PLLA-based BRS represent one of the main factors responsible for TLF and ScT (33–37). This has certainly driven the search for refined BRS platforms with different compositions and structural characteristics (38). A Mg-based coronary stent alloy reflects an interesting technology, since this metal has been shown to inhibit smooth cell proliferation and enhance endothelial integrity in experimental studies (39). Additionally, it showed faster resorption (95% at 12 months), improved crush resistance, less recoil after deployment, and better endothelial coverage over time compared to the PLLA-based and other BRS technologies (24).

Interestingly, we observed a steep increase in DoCE in two separate phases: between 0 to 6 months and 1 to 2 years after implantation, analogous to the Absorb™ BRS (10), with major contributors being acute ScT in the early phase and TLR due to restenosis in the later phase. Since the degradation process of the Magmaris™ BRS should be completed by 12 months, we cannot rule out for certain that progression of the underlying CAD contributed to the development of TLR and TVR in some cases.

The rather high rate of TLF was somewhat puzzling to us, since we followed a vigorous implantation protocol drawn from our broad experience with other scaffolds, particularly the Absorb™ device (26, 40). In this context, one also needs to be aware of the established mechanisms resulting in TLF with conventional metallic stents and BRS. Since early stent thrombosis usually indicates an underlying mechanical problem, such as undersizing, underexpansion, or impaired outflow, late and very late stent thrombosis, often results from incomplete strut coverage or plaque rupture secondary to neoatherosclerosis (41, 42). In contrast to our previous experience with the Absorb™ device, where early ScT usually occurred in underexpanded scaffolds, angiographic results did not suggest underexpansion in our patients with ScT (as highlighted in the case vignette with early ScT, Supplementary Figure 2) (26, 40).

On one hand, this might implicate that implantation of this device should not solely rely on angiographic measures, but rather intravascular imaging needs to be involved to ensure that the device is well-apposed and its integrity is not distorted (43). In fact, there is evidence that supports the liberal use of intravascular imaging whenever one considers implanting a BRS (43). On the other hand, it suggests that there may be some specific patient or lesion characteristics that detrimentally affect the performance of the Mg-BRS.

As previously mentioned, we observed early dismantling and device collapse in both the early and late stages (Figures 3, 4) and have been reported by other colleagues in the past (44–46). Additionally, it is important to recognize that the degradation process is not uniform throughout the full length of the scaffolded segment (Figure 4). Furthermore, we also found scaffold discontinuities and incomplete lesion coverage (Figure 4) (47–49). Interestingly, in one case, we encountered protruding struts up to 4 years after the Mg-BRS implantation (Figure 5). So far, the exact mechanisms leading to uncontrolled scaffold dismantling and discontinuities remain unclear. One may argue that the early disruption of the MgBRS potentially follows the vigorous postdilatation using high-pressures (24.2 ± 7.3 atm) and ultra-non-compliant balloons. This may have resulted in premature polymer hydration and scission of the ties that connect the amorphous phase with the crystalline phase. This, in turn, may have led to structural discontinuities and consequential loss of radial strength (50). Furthermore, high shear stress in the close vicinity of the rather thick struts may have promoted platelet activation and release of prothrombogenic molecules (e.g., adenosine diphosphate and thromboxane A2). Consequently, recirculation zones with low endothelial shear stress downstream of the strut increased the local concentration of activated platelets at the site of denuded endothelium in the absence of production of antithrombotic factors. All these factors together with a relatively low rate (7%) of intravascular imaging-guided implantation may have led to suboptimal scaffold implantation and enhanced risk of ScT (38, 51). Furthermore, in patients with early ScT, crossing with guide wires, aspiration catheters, and balloons proved often unusually challenging. This may indicate that disintegration and collapse of the scaffold were present in the first place, although not confirmed by intravascular imaging.

It is possible that treatment of unselected patients, including patients with MI, with the Mg-BRS, could have contributed to suboptimal implantation results in this study cohort. Especially, in patients with MI, the presence of thrombus, impaired coronary blood flow, and coronary vasospasms could hamper the optimal Mg-BRS implantation, which increases the risk for TLF and ScT. Nevertheless, the Magnesium-Based Resorbable Scaffold Versus Permanent Metallic Sirolimus-Eluting Stent in Patients With ST-Segment Elevation Myocardial Infarction (MAGSTEMI) trial, which compared the Mg-BRS and sirolimus-eluting metallic stents in patients with STEMI, underscored that there is no prohibitive signal for the use of this device in this clinical setting. However, the Mg-BRS implantation was related to lower angiographic efficacy and a higher rate of TLR compared to sirolimus-eluting DES in this trial (52).

Regarding antiplatelet management after BRS implantation, most of our patients received a potent second antiplatelet (ticagrelor or prasugrel) and therapy adherence was controlled by regular follow-up contacts. Thus, insufficient platelet inhibition as a possible cause for TLF seems unlikely.

This study has several limitations. First, this is an observational single-center study, which may limit the generalizability and does not allow drawing firm inferences. Second, the low adoption of intravascular imaging for the Mg-BRS implantation, particularly in more complex coronary lesions, could have led to a suboptimal scaffold sizing and implantation result. This, in turn, could have contributed to the relatively high rate of adverse events in our cohort. Third, we did not routinely perform angiographic follow-up examinations in our study cohort. In hindsight, we think that this might have been helpful in identifying some patients and lesions, which potentially carried a higher risk for adverse outcomes and, thus, could have benefited from specific therapeutic measures (e.g., intensified or prolonged DAPT). But, there are only limited data supporting such an approach as of yet (53). Fourth, only four operators were involved in this study. Nonetheless, they were all the experienced interventionalists (> 10 years of experience) with large expertise in the use of BRS (40). Fifth, we were, unfortunately, not able to perform intravascular imaging among all the cases presenting with DoCE, even though our standard of practice usually involves intravascular imaging (preferentially OCT) in cases with scaffold failure or thrombosis. Of note, we encountered cases with the Mg-BRS-related ScTs where it was nearly impossible to cross the lesion and restore flow. Finally, we have, unfortunately, lost five patients during the follow-up period (all beyond 2 years of the follow-up period).

Our data support the latest recommendation by the European Society of Cardiology (ESC) guidelines, which state that BRS should only be used in highly selected patients or in the trial setting (53). From our side, we certainly need to acknowledge that novel PCI devices, especially scaffolds, should be introduced cautiously to one's practice. Above all, one may start with simple lesions. With regard to the Mg-BRS, we think that there is a need for more dedicated trials to guide patient and lesion selection, but also to understand the exact mechanism leading to the failure of this scaffold.

## Conclusion

In contrast to earlier studies, we found a relatively high rate of DoCE in an all-comer cohort treated with the Mg-BRS Magmaris™. Of note, most adverse events were observed within 24 months after implantation and very few TLFs occurred thereafter. Regarding the patterns of TLF, we encountered scaffold collapse and uncontrolled dismantling. This may implicate that this metal-based BRS requires further investigation and may only be used in highly selected cases.

## Data Availability Statement

The raw data supporting the conclusions of this article will be made available by the authors, without undue reservation.

## Ethics Statement

The studies involving human participants were reviewed and approved by Ethikkommission Nordwest- und Zentralschweiz (EKNZ). The Ethics Committee waived the requirement of written informed consent for participation. Written informed consent was obtained from the individual(s) for the publication of any potentially identifiable images or data included in this article.

## Author Contributions

MB, RK, GS, and FC: organization and project planing. MB, MM, DA, AA-T, GC, TS, GT, and FC: data collection. MB, MM, AA-T, and TS: data analysis. MB, MM, and FC: drafting of the manuscript. All authors: reviewing of the manuscript. All authors contributed to the article and approved the submitted version.

## Conflict of Interest

MB has received speaker and/or consultant fees from Abbott Vascular, Amgen, AstraZeneca, Abiomed, Amgen, Bayer, Daiichi Sankyo, Mundipharma, and SIS Medical. RK has received institutional grants from Abbott, Biosense Webster, Biotronik, Boston Scientific, Medtronic, and SIS Medical and serves as a consultant for Biosense Webster and Biotronik. FC has received speaker and consulting fees from Abbott Vascular, Abiomed, and SIS Medical. The remaining authors declare that the research was conducted in the absence of any commercial or financial relationships that could be construed as a potential conflict of interest.

## Publisher's Note

All claims expressed in this article are solely those of the authors and do not necessarily represent those of their affiliated organizations, or those of the publisher, the editors and the reviewers. Any product that may be evaluated in this article, or claim that may be made by its manufacturer, is not guaranteed or endorsed by the publisher.

## Supplementary Material

Supplementary Figure 1 Case series of three patients presenting with acute and very late scaffold thrombosis (ScT). **(A)** Acute ScT of the mid right coronary artery (RCA) 10 days after implantation of the 1 Magmaris bioresorbable scaffold (BRS). After predilatation with a non-compliant (NC) balloon (2.0 × 15 mm, 20 atm), 3 DES (2.5 × 15 mm, 3.5 × 40 mm, and 3.5 × 48 mm) were implanted. **(B)** Very late ScT of the proximal left anterior descending (LAD) 380 days after implantation of 1 Magmaris BRS. After thrombectomy and predilatation with a NC balloon (2.0 × 15 mm, 14 atm), 1 DES was implanted (3.5 × 18 mm,18 atm) **(C)** Acute ScT of the mid LAD 5 days after implantation of the 1 Magmaris BRS. After predilatation with a super NC balloon (2.5 × 20 mm, 35 atm), 3 DES were implanted (2.25 × 30 mm, 2.5 × 13 mm, and 3.0 × 15 mm).

Supplementary Figure 2 Angiographic presentation of a patient with the Mg-BRS-related ScT. **(A–C)** Patient undergoing percutaneous coronary intervention (PCI) of a significant lesion of the mid LAD artery (90% stenosis, *arrow*): **(A)** Initial angiogram (^*^lesion before PCI). **(B)** Final angiogram after the Mg-BRS implantation and postdilatation (^†^lesion after treatment). **(C)** Angiogram at presentation with ScT (^‡^). Mg-BRS, magnesium-based sirolimus-eluting bioresorbable scaffold; ScT, scaffold thrombosis.

Supplementary Table 1 Independent predictors for DoCE. Data are mean reported as hazard ratio (HR) and 95% confidence interval (CI). ACS, acute coronary syndrome; CABG, coronary artery bypass graft; CTO, chronic total occlusion; DoCE, device-oriented composite endpoint; ISR, in-stent restenosis; IV, intravascular; LVEF, left ventricular ejection fraction; MI, myocardial infarction. **P*-values were based on Cox regression analysis.

Supplementary Table 2 Comparison of the Lucerne MAGMARIS registry and the BIOSOLVE-IV registry (13, 32). Data are mean (standard deviation) or number (percentage), as appropriate. CCS, chronic coronary artery syndrome; CTO, Chronic total occlusion; ISR, Instent-restenosis; MI, Myocardial infarction; N/A, Not applicable; NSTEMI, Non-ST-segment elevation myocardial infarction; STEMI, ST-segment elevation myocardial infarction; UA, Unstable angina. ^*^The BIOSOLVE-IV study (ClinicalTrials.gov: NCT02817802) represents an international, single arm, multicenter registry, which aimed to assess the performance and long-term safety of the Magmaris™ scaffold. It was conducted in more than 80 centers in 23 countries in Europe, Asia, Africa, and Australia/New Zealand. The main inclusion criteria represented a maximum of two single de novo lesions in two different major epicardial vessels, lesion length ≤ 21 mm, target lesion stenosis >50% and <100%, TIMI flow ≥1, and reference vessel diameter between 2.7 and 3.7 mm. The main exclusion criteria were left main disease, instent-restenosis, acute STEMI, bifurcation lesions, and unsuccessful pre-dilatation.
