# Supplementary figures and images for: Long-Term Outcomes After Implantation of Magnesium-Based Bioresorbable Scaffolds—Insights From an All-Comer Registry
Source: Front Cardiovasc Med. 2022 Apr 14;9:856930. doi: 10.3389/fcvm.2022.856930 (PMC9046914; doi:10.3389/fcvm.2022.856930)

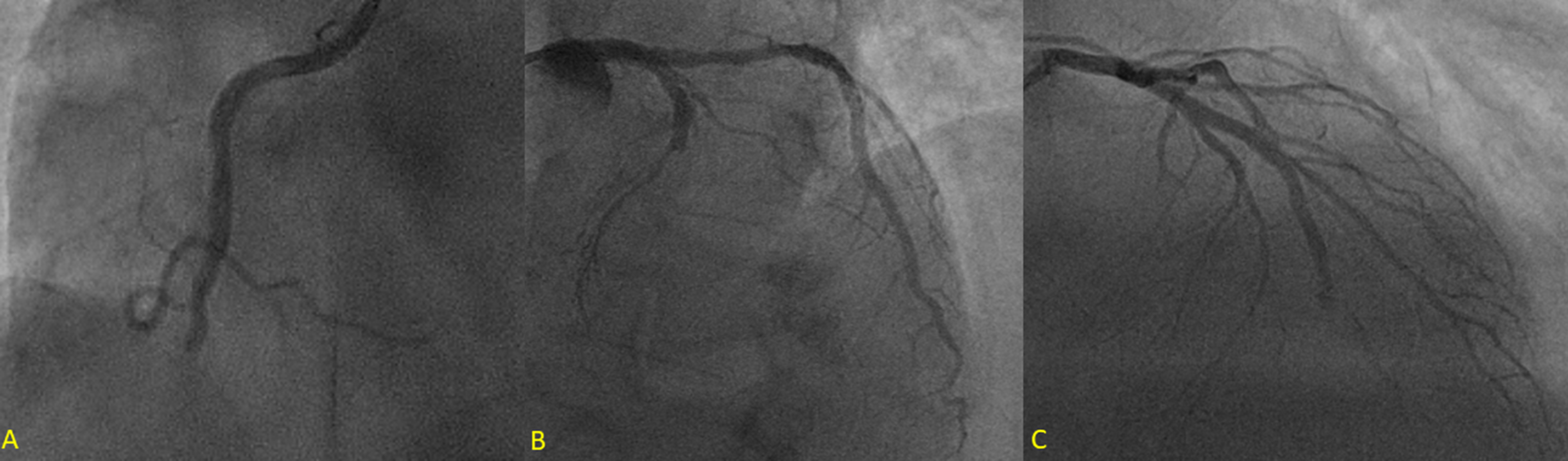

Supplement: Supplementary Figure 1 — Case series of three patients presenting with acute and very late scaffold thrombosis (ScT). (A) Acute ScT of the mid right coronary artery (RCA) 10 days after implantation of the 1 Magmaris bioresorbable scaffold (BRS). After predilatation with a non-compliant (NC) balloon (2.0 × 15 mm, 20 atm), 3 DES (2.5 × 15 mm, 3.5 × 40 mm, and 3.5 × 48 mm) were implanted. (B) Very late ScT of the proximal left anterior descending (LAD) 380 days after implantation of 1 Magmaris BRS. After thrombectomy and predilatation with a NC balloon (2.0 × 15 mm, 14 atm), 1 DES was implanted (3.5 × 18 mm,18 atm) (C) Acute ScT of the mid LAD 5 days after implantation of the 1 Magmaris BRS. After predilatation with a super NC balloon (2.5 × 20 mm, 35 atm), 3 DES were implanted (2.25 × 30 mm, 2.5 × 13 mm, and 3.0 × 15 mm). [file Image_1.tiff]

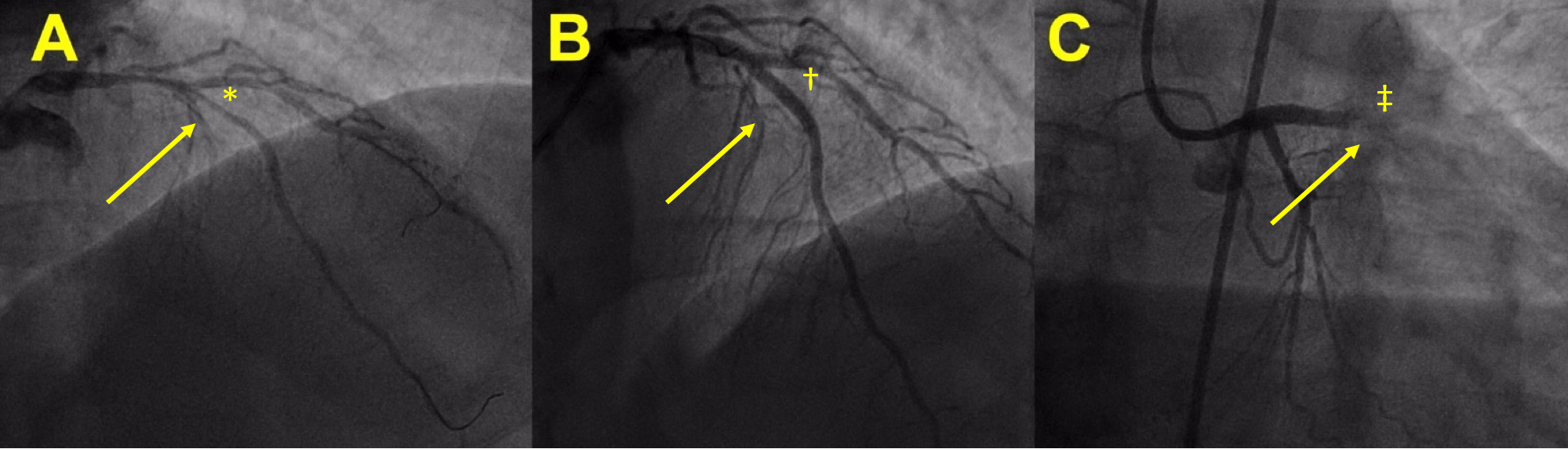

Supplement: Supplementary Figure 2 — Angiographic presentation of a patient with the Mg-BRS-related ScT. (A–C) Patient undergoing percutaneous coronary intervention (PCI) of a significant lesion of the mid LAD artery (90% stenosis, arrow): (A) Initial angiogram (*lesion before PCI). (B) Final angiogram after the Mg-BRS implantation and postdilatation (†lesion after treatment). (C) Angiogram at presentation with ScT (‡). Mg-BRS, magnesium-based sirolimus-eluting bioresorbable scaffold; ScT, scaffold thrombosis. [file Image_2.tiff]
