# Supplementary material for: Long-Term Outcomes After Implantation of Magnesium-Based Bioresorbable Scaffolds—Insights From an All-Comer Registry
Source: Front Cardiovasc Med. 2022 Apr 14;9:856930. doi: 10.3389/fcvm.2022.856930 (PMC9046914; doi:10.3389/fcvm.2022.856930)
Supplement: Supplementary Table 1 — Independent predictors for DoCE. Data are mean reported as hazard ratio (HR) and 95% confidence interval (CI). ACS, acute coronary syndrome; CABG, coronary artery bypass graft; CTO, chronic total occlusion; DoCE, device-oriented composite endpoint; ISR, in-stent restenosis; IV, intravascular; LVEF, left ventricular ejection fraction; MI, myocardial infarction. *P-values were based on Cox regression analysis. [file Table_1.docx]

**Supplemental table 1** Independent predictors for DoCE

| Variable | HR | (95% CI) | p-value^*^ |
| --- | --- | --- | --- |
| Age | 0.99 | 0.95-1.0 | 0.76 |
| ACS | 0.71 | 0.23-2.14 | 0.55 |
| LVEF | 0.97 | 0.91-1.00 | 0.41 |
| Creatinine | 0.99 | 0.95-1.00 | 0.48 |
| Hypertension | 1.47 | 0.32-6.61 | 0.62 |
| Dyslipidemia | 1.95 | 0.46-8.33 | 0.37 |
| Diabetes | 1.24 | 0.16-9.46 | 0.84 |
| Smoking | 1.51 | 0.23-1.00 | 0.67 |
| Previous MI | 0.66 | 0.09-4.31 | 0.67 |
| Previous CABG | 2.20 | 0.2-2.32 | 0.52 |
| ISR | 1.27 | 0.59-1.94 | 0.95 |
| Bifurcation | 1.33 | 0.25-7.11 | 0.73 |
| CTO | 0.20 | 0.25-1.54 | 0.12 |
| Severe calcification | 1.47 | 0.58-3.73 | 0.42 |
| Lesion diameter | 1.25 | 0.08-1.83 | 0.93 |
| Lesion length | 1.05 | 0.91-1.22 | 0.48 |
| Use of IV imaging | 0.06 | 0.02-1.61 | 0.09 |
| Scaffold inflation pressure | 0.99 | 0.9-1.00 | 0.83 |
| Post-dilatation pressure | 1.02 | 0.94-1.12 | 0.56 |
| Hybrid lesion treatment | 0.66 | 0.2-2.11 | 0.49 |

Data are mean reported as hazard ratio (HR) and 95% confidence interval (CI). ACS= acute coronary syndrome; CABG: coronary artery bypass graft; CTO= chronic total occlusion; DoCE = device-oriented composite endpoint; ISR= in-stent restenosis; IV= intravascular; LVEF= left ventricular ejection fraction; MI = myocardial infarction.

^*^ P values were based on Cox regression analysis.
