# Supplementary material for: Long-Term Outcomes After Implantation of Magnesium-Based Bioresorbable Scaffolds—Insights From an All-Comer Registry
Source: Front Cardiovasc Med. 2022 Apr 14;9:856930. doi: 10.3389/fcvm.2022.856930 (PMC9046914; doi:10.3389/fcvm.2022.856930)
Supplement: Supplementary Table 2 — Comparison of the Lucerne MAGMARIS registry and the BIOSOLVE-IV registry (13, 32). Data are mean (standard deviation) or number (percentage), as appropriate. CCS, chronic coronary artery syndrome; CTO, Chronic total occlusion; ISR, Instent-restenosis; MI, Myocardial infarction; N/A, Not applicable; NSTEMI, Non-ST-segment elevation myocardial infarction; STEMI, ST-segment elevation myocardial infarction; UA, Unstable angina. *The BIOSOLVE-IV study (ClinicalTrials.gov: NCT02817802) represents an international, single arm, multicenter registry, which aimed to assess the performance and long-term safety of the Magmaris™ scaffold. It was conducted in more than 80 centers in 23 countries in Europe, Asia, Africa, and Australia/New Zealand. The main inclusion criteria represented a maximum of two single de novo lesions in two different major epicardial vessels, lesion length ≤ 21 mm, target lesion stenosis >50% and <100%, TIMI flow ≥1, and reference vessel diameter between 2.7 and 3.7 mm. The main exclusion criteria were left main disease, instent-restenosis, acute STEMI, bifurcation lesions, and unsuccessful pre-dilatation. [file Table_2.docx]

**Supplemental Table 2** Comparison of the Lucerne MAGMARIS Registry and the BIOSOLVE-IV Registry (13, 32).

|  | ***Lucerne MAGMARIS***  ***Registry*** | *BIOSOLVE-IV Registry^*^* |
| --- | --- | --- |
| **Patient characteristics:** | ***No. patients***  *(n=84)* | ***No. Patients***  *(n=1075)* |
| Age (years ±SD) | 62±11 | 61±10 |
| Males, n (%) | 63 (75) | 806 (75) |
| *Presentation, n (%)* |  |  |
| CCS | 28 (34) | 869 |
| UA/ NSTEMI | 34 (40) | 206 |
| STEMI | 22 (26) | N/A |
| Diabetes mellitus n (%) | 10 (12) | 228 (21) |
| Previous MI, n (%) | 19 (23) | 219 (20) |
| **Lesion characteristics:** | ***No. lesions***  *(n=101)* | ***No. lesions***  *(n=1121)* |
| AHA/ACC classification type B2/C, n (%) | 35 (35) | 170 (15) |
| Bifurcation lesions, n (%) | 16 (16) | 57 (5) |
| CTO, n (%) | 3 (3) | N/A |
| ISR, n (%) | 8 (8) | N/A |
| **Procedural characteristics:** | ***No. lesions***  *(n=101)* | ***No. lesions***  *(n=1121)* |
| Pre-dilatation performed, n (%) | 101 (100) | 1,118 (99.7) |
| Mean device length (mm ±SD) | 22.1±3.3 | 19.6 ± 3.9 |
| Mean device diameter (mm ±SD) | 3.25±0.25 | 3.2 ± 0.3 |
| Post-dilatation, n (%) | 98 (97) | 1,081 (96.4) |
| Maximal post-dilatation pressure (atm ±SD) | 24.2±7.3 | 17.1 ± 3.3 |
| Hybrid lesion treatment, n (%) | 28 (8) | N/A |
| Fenestration of side branch, n (%) | 11 (11) | N/A |
| Device success, n (%) | 101 (100) | 1,129 (97.3) |
| Procedure success, n (%) | 101 (100) | 1,063 (98.9) |

Data are mean (standard deviation) or number (percentage), as appropriate. CCS= chronic coronary artery syndrome; CTO = Chronic total occlusion; ISR = Instent-restenosis; MI = Myocardial infarction; N/A = Not applicable; NSTEMI = Non-ST-segment elevation myocardial infarction; STEMI = ST-segment elevation myocardial infarction; UA = Unstable angina.

*^*^* The *BIOSOLVE-IV study* ([ClinicalTrials.gov](http://clinicaltrials.gov): NCT02817802) represents an international, single arm, multicenter registry, which aimed to assess the performance and long-term safety of the Magmaris™ scaffold. It was conducted in more than 80 centers in 23 countries in Europe, Asia, Africa, and Australia/New Zealand. The main inclusion criteria represented a maximum of two single de novo lesions in two different major epicardial vessels, lesion length ≤21 mm, target lesion stenosis >50% and <100%, TIMI flow ≥1, and reference vessel diameter between 2.7 and 3.7 mm. The main exclusion criteria were left main disease, instent-restenosis, acute STEMI, bifurcation lesions, and unsuccessful pre-dilatation.
